# Supplementary material for: CDK4/6 or MAPK blockade enhances efficacy of EGFR inhibition in oesophageal squamous cell carcinoma
Source: Nat Commun. 2017 Jan 6;8:13897. doi: 10.1038/ncomms13897 (PMC5227099; doi:10.1038/ncomms13897)
Supplement: Supplementary Information — Supplementary Figures and Supplementary Tables [file ncomms13897-s1.pdf]

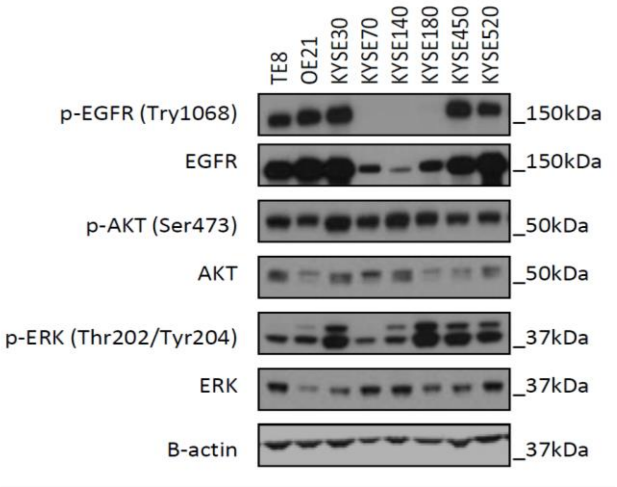

Supplementary Figure 1. Basal level EGFR across a panel of ESCC lines.
Immunoblots demonstrate the expression of phosphorylated and total EGFR as well as their downstream effectors across a panel of ESCC cell lines. Protein was harvested 24 hours after plating at the desired densities. Cells were cultured in full serum-containing medium.

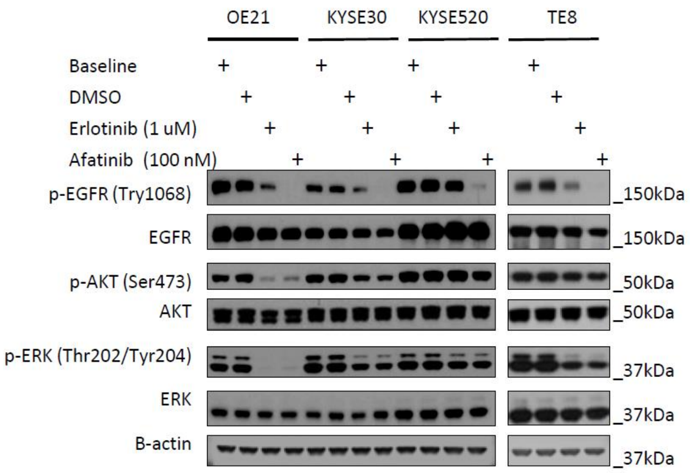

Supplementary Figure 2. Downstream signaling changes predict the response to EGFR inhibition in EGFR-amplified ESCC cell line models.

OE21, KYSE30, KYSE520 and TE8 cells were treated with 1uM erlotinib or 100nM afatinib for 6 hours and EGFR, AKT and ERK phosphorylation (p) was assessed.

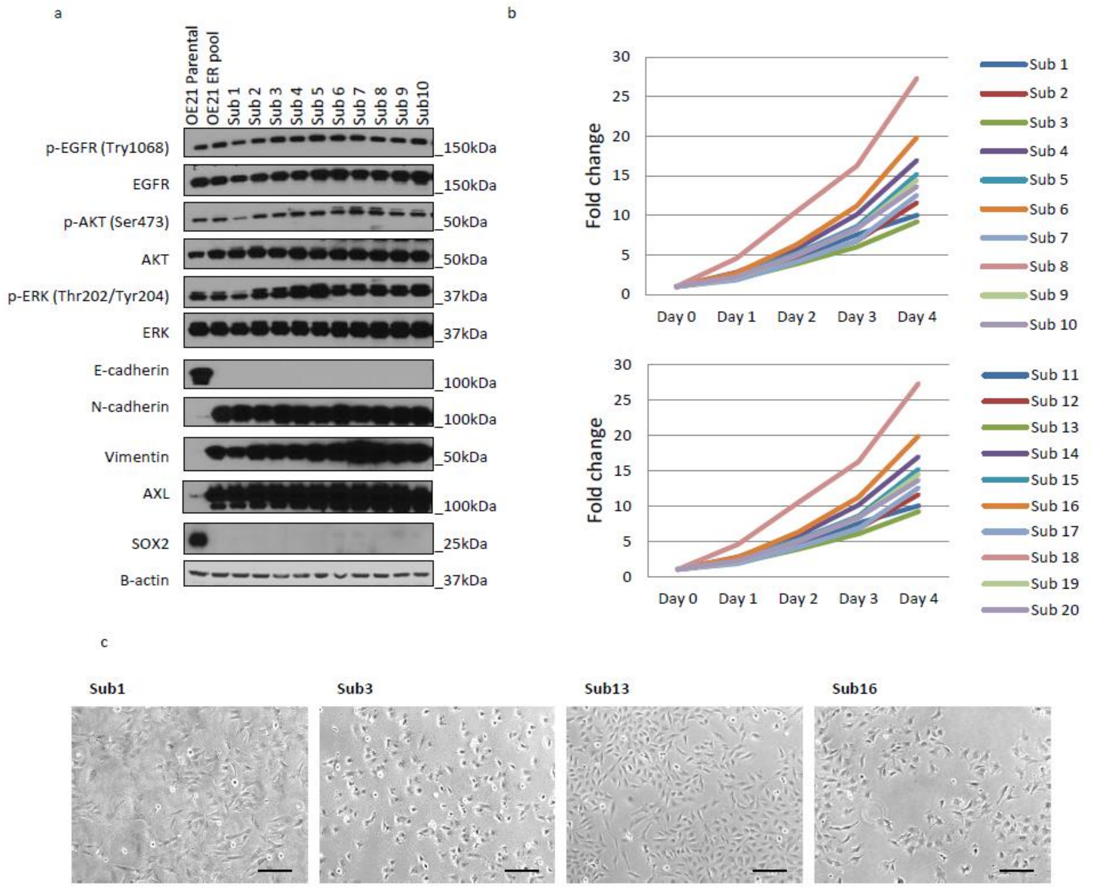

Supplementary Figure 3. Heterogeneous resistance phenotypes in individual subclones of erlotinib resistant OE21.

(a)Basal level of different OE21 subclones established following development of acquired resistance to EGFR inhibition. Immunoblots show EGFR, ERK, AKT phosphorylation, SOX2, AXL and EMT-related markers. (b)different growth rate of subclones. (c) Representative images of subclones demonstrating different morphology under inverted microscopy. Scale bar, 100 µm.

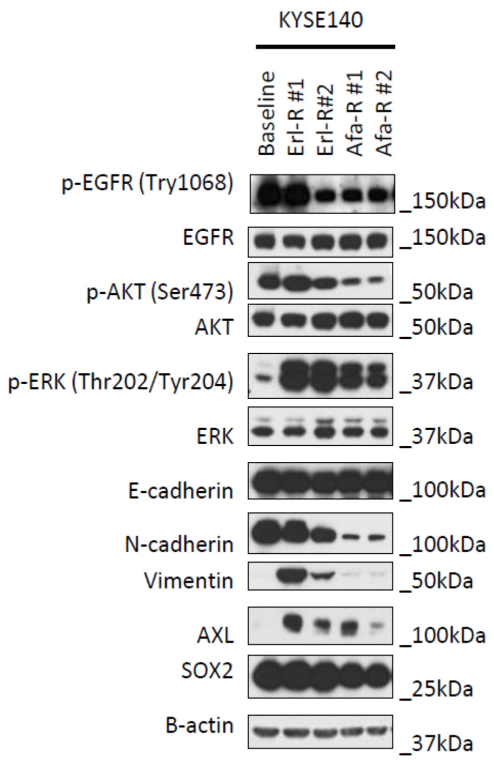

Supplementary Figure 4. Mechanism of EGFR-resistant KYSE140.

Immunoblot measurement of candidate EMT markers of KYSE140 parental, erlotinib-resistant, and afatinib-resistant cell lines.

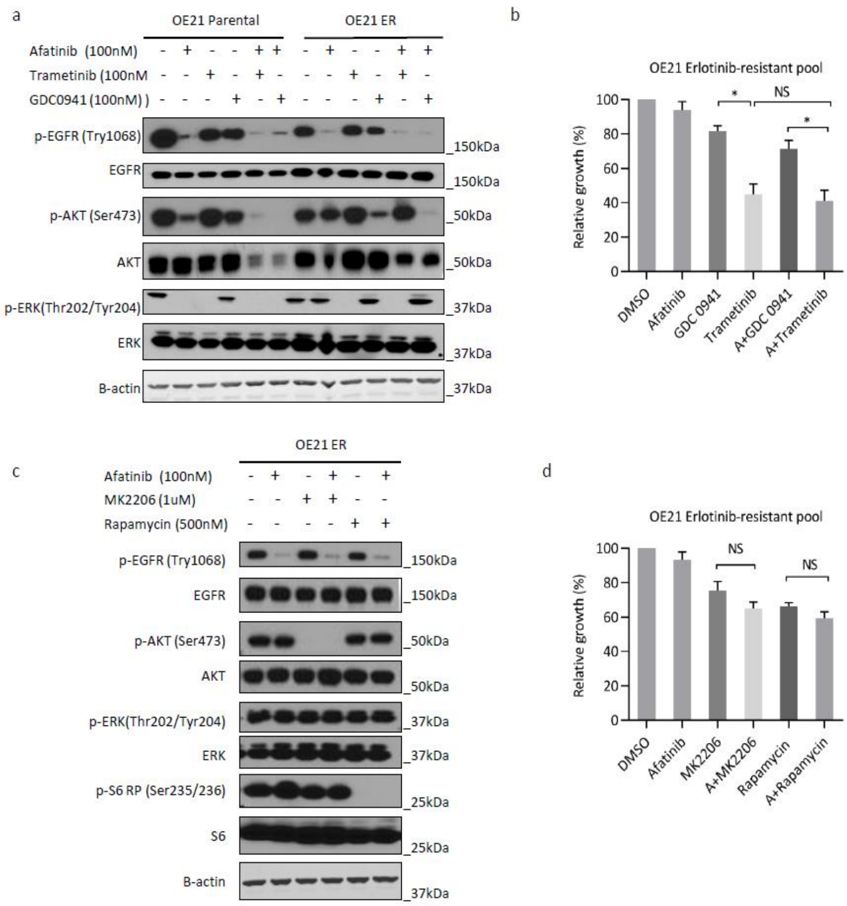

Supplementary Figure 5. OE21 erlotinib resistant cells are modest response or resistant to MEK, PI3K, AKT or mTOR inhibitors with afatinib.

OE21 ER cells were treated with AKT inhibitor MK2206 or mTOR inhibitor Rapamycin either alone or in combination with afatinib.(a) Immunoblots evaluating the biochemical effects of treatment with 100nM afatinib and 100nM MEK or 100nM PI3K inhibitors. Cells were harvested 6 hours after drug treatment. (c) Immunoblots evaluating the biochemical effects of treatment with 100nM afatinib and 1uM MK2206 or 500nM Rapamycin. Cells were harvested 6 hours after drug treatment. (b) (d) In vitro 72hrs growth inhibition by two small-molecule inhibitors either alone or in combination in the indicated doses. Data represent drug treatment conditions and relative cell viability compared with that of DMSO-treated control. Each data point represents the mean±SD. All experiments were performed in triplicate for each condition and repeated at least twice.Student *t*-test was used for statistical analysis. (* $p<0.05$)

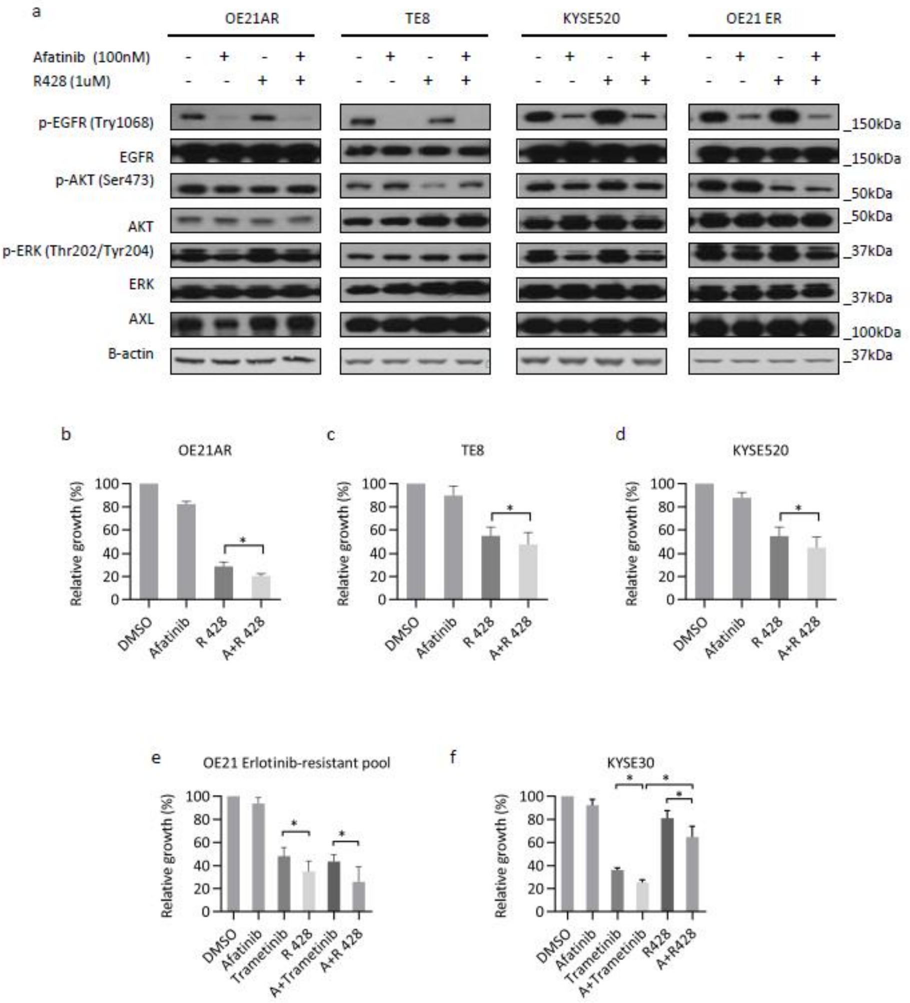

Supplementary Figure 6. Response of OE21 erlotinib-resistant, afatinib-resistant cells, TE8 and KYSE520 to AXL inhibitor R428.

(a) Immunoblots evaluating the biochemical effects of treatment with 100nM afatinib and 1uM R428. Cells were harvested 6 hours after drug treatment. (b-e) Cells were treated with AXL inhibitor R428 either alone or in combination with afatinib in the indicated doses for 72hrs. Data represent drug treatment conditions and relative cell viability compared with that of DMSO-treated control (f) KYSE30 cells were treated with MEK inhibitor trametinib or AXL inhibitor R428 either alone or in combination with afatinib for 72hrs. Each data point represents the mean ± SD. All experiments were performed in triplicate for each condition and repeated at least twice. Student *t*-test was used for statistical analysis. * p<0.05)

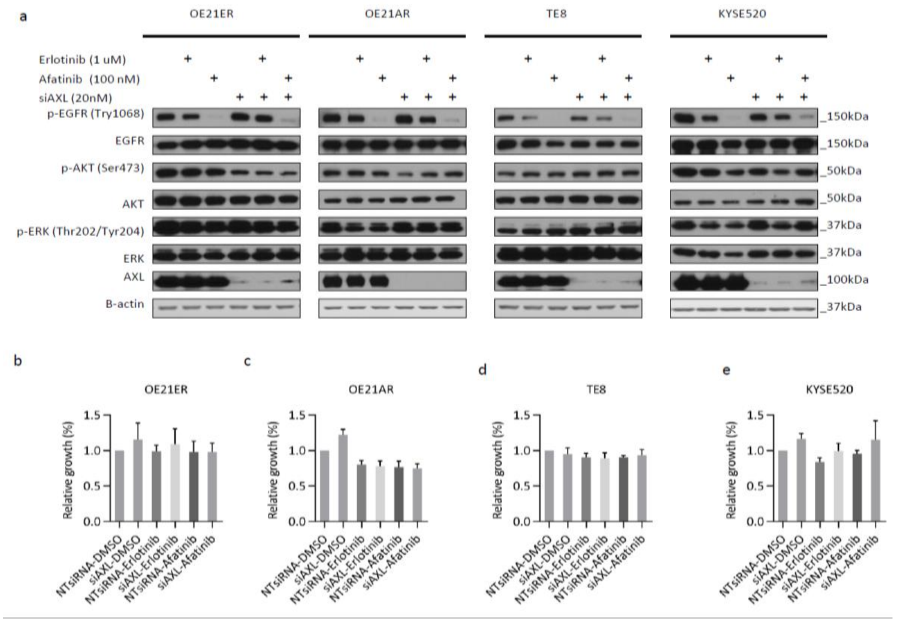

Supplementary Figure 7. Response of OE21 erlotinib, afatinib resistant cells, TE8 and KYSE520 to EGFR inhibition and knock down AXL.

(a) Western blot showing biochemical effects of AXL knockdown and EGFR inhibition. Cells were harvested after 48hours transfection in addition to 6 hours drug treatment (b) Cell viability (3days) of cells subjected to AXL knockdown and treated as indicated. All experiments were performed in triplicate for each condition and repeated at least twice. Each data point represents the mean ± SD.

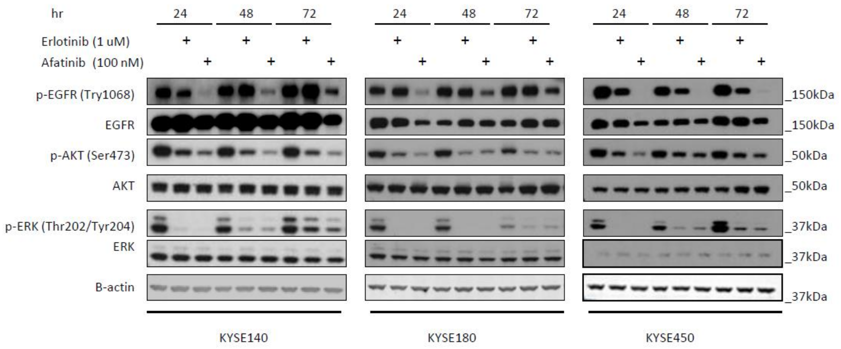

Supplementary Figure 8. Activation of ERK as adaptive response to EGFR inhibition in KYSE140, KYSE180 and KYSE450 cells.

Immunoblot analysis of protein phosphorylation extracts at distinct timepoints from KYSE140 cells treated with 1uM erlotinib or 100nM afatinib.

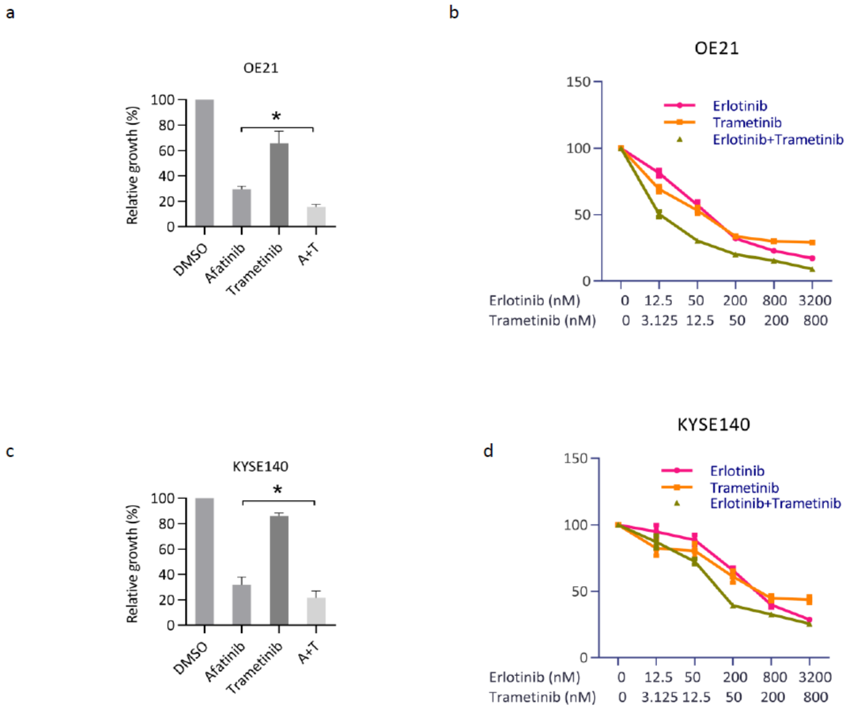

Supplementary Figure 9. Effects of trametinib treatment in combination with afatinib in OE21 and KYSE140 cells.

Cell viability assays from OE21 and KYSE140 cells following exposure to control (DMSO), afatinib, trametinib, or combinations at the indicated concentration for 72 hours. (a) The data were analyzed by CompuSyn software, and the summary growth histogram, dose-effect curve, CI values were shown. Combination index analysis showed that a combination index of 1 reflects additive effects, wherease values greater than and less than 1 indicate antagonism and synergy, respectively.

(b) OE21 and KYSE140 treated in vitro with either 100nM afatinib or 100nM trametinib alone or in combination. All data are expressed as the percentage of growth relative to that of DMSO-treated control cells. Each data point represents the mean ± SD. All experiments were performed in triplicate for each condition and repeated at least twice. Student *t*-test was used for statistical analysis. * $p<0.05$)

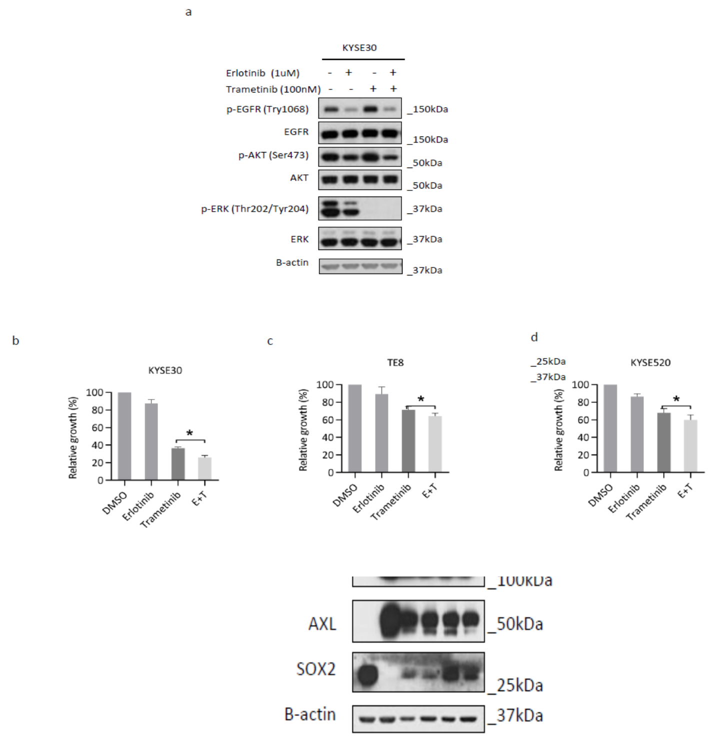

Supplementary Figure 10. Mechanism of EGFR/MEK inhibitors-resistance in OE21 cells. Immunoblot measurement of candidate EMT markers of OE21 parental, erlotinib+trametinib-resistant, and afatinib+trametinib-resistant cell lines.

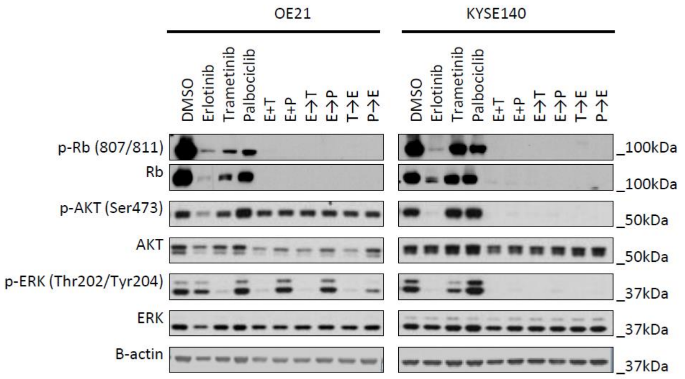

Supplementary Figure 11. The MEK inhibitor trametinib reverses intrinsic resistance to EGFR inhibition in Ras-mutation KYSE30 cell line, but not TE8 and KYSE520 lines.

(a) Immunoblot demonstrates biochemical impacts in KYSE30 cells of erlotinib alone or in combination with trametinib. Cells were harvested 6 hours after each treatment. (b-d) growth inhibition of EGFR amplified-ESCC cell lines KYSE30, TE8 and KYSE520 treated with either erlotinib or trametinib alone or by a combination of both. All data are expressed as the percentage of growth relative to that of DMSO-treated control cells. KYSE30 cells possess endogenouse *HRAS* mutation (Q61L) together with *EGFR* amplification. Each data point represents the mean±SD. All experiments were performed in triplicate for each condition and repeated at least twice. Student *t*-test was used for statistical analysis. (* $p<0.05$).

Supplementary Figure 12. Biochemical effects of CDK4/6 blockade with EGFR inhibition in in EGFR-amplified ESCC cell lines.

Immunoblots of protein lysates from OE21 and KYSE140 treated with labelled inhibitors alone or in combinations.

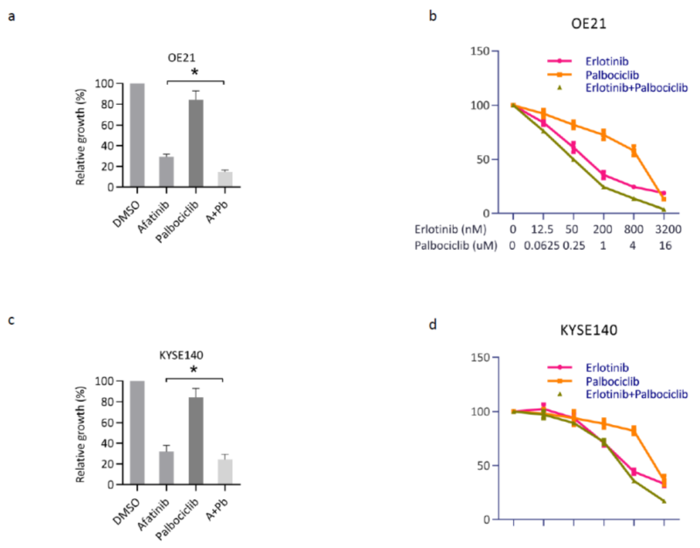

Supplementary Figure 13. Effects of palbociclib treatment in combination with afatinib in OE21 and KYSE140 cells.

Cell viability assays from OE21 and KYSE140 cells following exposure to control (DMSO), afatinib, palbociclib, or combinations at the indicated concentration for 72 hours. (a) OE21 and KYSE140 treated in vitro with either 100nM afatinib or 1uM palbociclib alone or in combination. All data are expressed as the percentage of growth relative to that of DMSO-treated control cells. (b) The data were analyzed by CompuSyn software, and the summary growth histogram, dose-effect curve, CI values were shown. Combination index analysis showed that a combination index of 1 reflects additive effects, wherease values greater than and less than 1 indicate antagonism and synergy, respectively. Each data point represents the mean±SD. (student *t*-test was used for statistical analysis. * p<0.05).

a

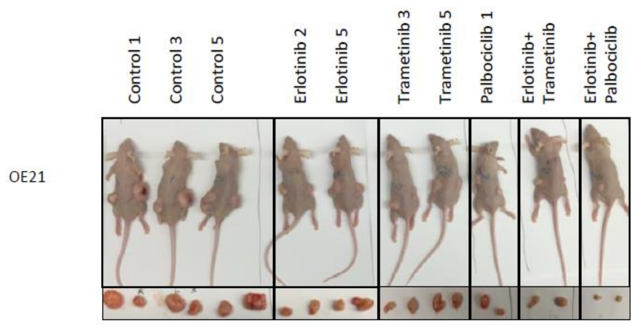

b

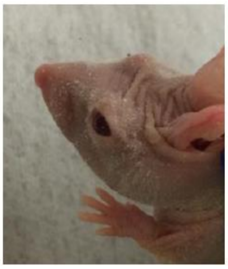

Supplementary Figure 14. Treatment of OE21 xenografts with EGFR inhibition alone or in combination with CDK4/6 or MEK inhibition.

(a) Representative photographs of mice in each group taken 28 days after treatment. (b) Images of tumors from OE21 xenografts when nude mice sacrificed. (c) Presence of skin rash in a mouse treated with MEK/EGFR combination therapy.

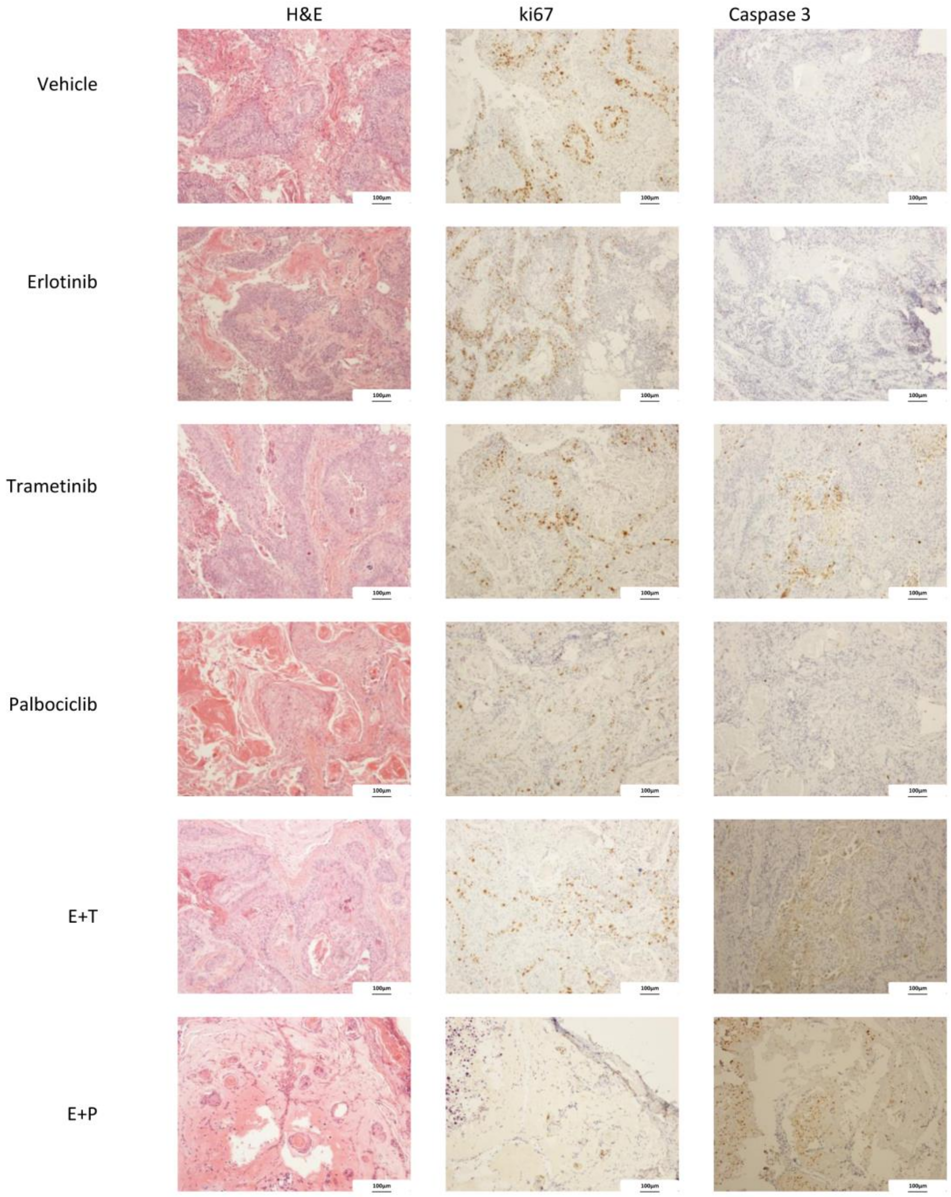

Supplementary Figure 15. Pathology analyze of OE21 xenografts tumors.

The proliferation marker (Ki67), and apoptosis marker (caspase3) were analyzed with IHC. Scale bar, 100 μm.

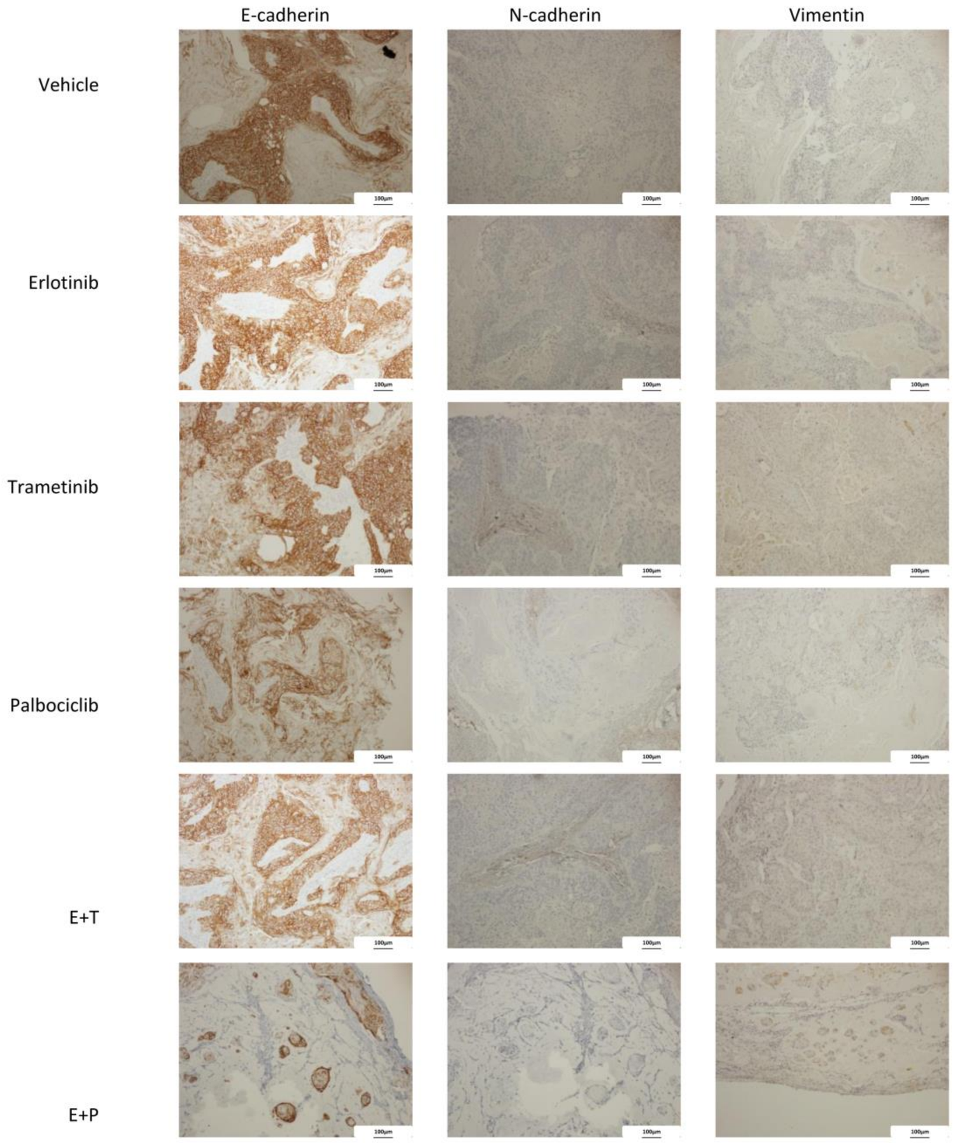

Supplementary Figure 16. Pathology analyze of OE21 xenografts tumors.

The EMT marker E-cadherin, N-cadherin and Vimentin were analyzed with IHC. Scale bar, 100 µm.

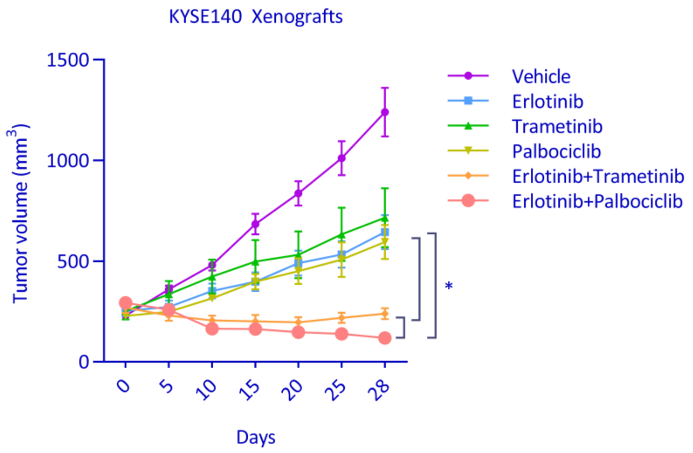

Supplementary Figure 17. CDK4/6 inhibition improves erlotinib response in ESCC xenografts.

Curves plot the growth of OE21 xenograft tumors were treated with vehicle control, erlotinib, palbociclib, or erlotinib+palbociclib. (mean ± SEM, 3-5 mice per condition).

Fig 1a

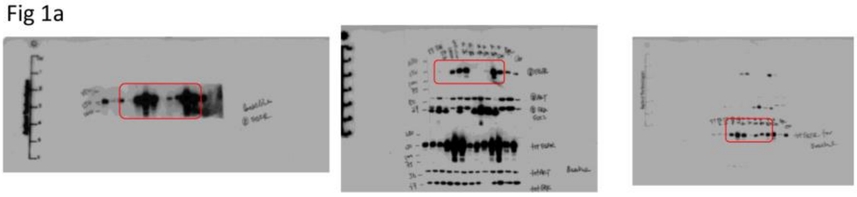

Fig 1d

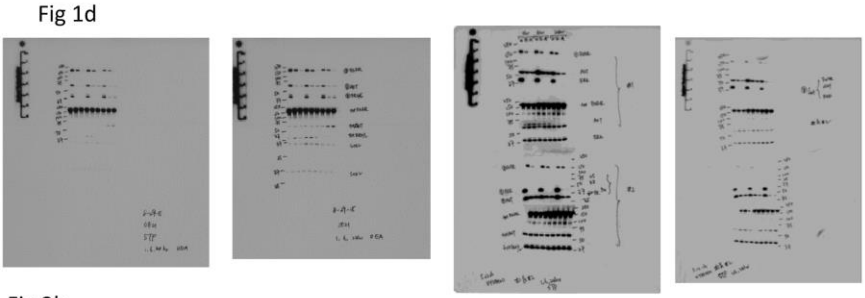

Fig 2b

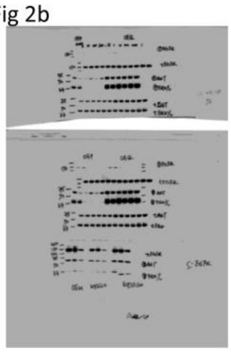

Fig 2d

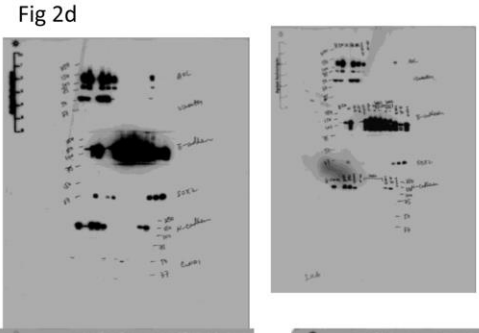

Fig 2e

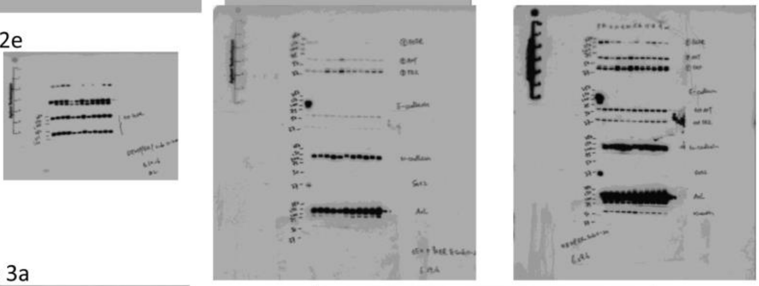

Fig 3a

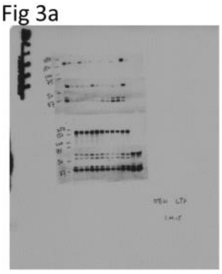

Fig 3b

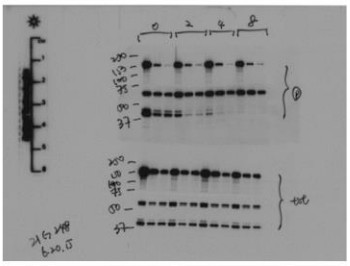

Supplementary Figure 18. Full images of cropped blots presented in other figures.

Supplementary Table 1. IC50 of erlotinib and afatinib in a panel of ESCC cell lines

**Cell line**	**Erlotinib (μM)**	**Afatinib (μM)**
**TE8**	10.74	1.95
**OE21**	0.19	0.02
**KYSE30**	15.86	1.67
**KYSE70**	3.61	0.42
**KYSE140**	0.31	0.05
**KYSE180**	0.40	0.004
**KYSE450**	0.33	0.04
**KYSE520**	10.98	3.54

Supplementary Table 2. Selectively Genomic Alterations in a panel of ESCC cell line models

Cell line	Genomic alteration
**TE8**	EGFR amplification
**OE21**	EGFR amplification, CCND1 amplification, p16 deletion
**KYSE30**	EGFR amplification, H-ras Q61L
**KYSE70**	EGFR T68fs
**KYSE140**	EGFR amplification, p16 deletion
**KYSE180**	CCND1 amplification
**KYSE450**	EGFR amplification, EGFR S768I
**KYSE520**	EGFR amplification
